# Hygiene Practices and Early Childhood Development in the East Asia-Pacific Region: A Cross-Sectional Analysis

**DOI:** 10.3390/ijerph20042798

**Published:** 2023-02-04

**Authors:** Fanny Petermann-Rocha, Nirmala Rao, Manya Bala, Monika Parshad-Asnani, Anthony Sifuna, Aisha Yousafzai, Frederick K. Ho, Patrick Ip

**Affiliations:** 1 Centro de Investigación Biomédica, Facultad de Medicina, Universidad Diego Portales, Santiago 8370068, Chile; 2 BHF Cardiovascular Research Centre, School of Cardiovascular and Metabolic Health, University of Glasgow, Glasgow G12 8TA, UK; 3 Faculty of Education, The University of Hong Kong, Hong Kong; 4 Sickle Cell Unit, Caribbean Institute for Health Research, The University of The West Indies, Kingston 7, Jamaica; 5 Department of Medical Biochemistry, Masinde Muliro University of Science and Technology, Kakamega 50100, Kenya; 6 Harvard T.H. Chan School of Public Health, Harvard University, Cambridge, MA 02115, USA; 7 School of Health and Wellbeing, University of Glasgow, Glasgow G12 8RZ, UK; 8 Department of Paediatrics and Adolescent Medicine, The University of Hong Kong, Hong Kong

**Keywords:** hand hygiene, oral hygiene, child, growth and development, East Asia-Pacific region

## Abstract

Poor hygiene might be a risk factor for early childhood development (ECD). This study investigated the associations of three hygiene practices (‘wash hands before a meal,’ ‘wash hands after going to the toilet,’ and ‘brush teeth’), separately and combined, with ECD. Six thousand six hundred ninety-seven children (4 [0.8] years) from the East Asia-Pacific Early Child Development Scales validation study were included in this cross-sectional analysis. The hygiene variables were recoded to have comparable values as ‘always,’ ‘sometimes,’ and ‘never.’ These variables were then grouped to create combined categories. The binary outcome variables, poor ECD, were defined as a score < age-specific 25th centile. Modified Poisson regression models were used to analyse the associations. Data collection was performed between 2012 and 2014, and the analyses were conducted in April 2022. Compared with children who ‘always’ washed their hands before a meal, those who did it ‘sometimes’ (Prevalence Ratio [PR]: 1.30 [95% CI: 1.16–1.46]) or ‘never’ (PR: 1.35 [1.18–1.55]) had a higher likelihood of poorer overall development. Comparable results were identified for the other two hygiene practices and the other four domain-specific outcomes (*p* < 0.05). Compared with children who always followed the three hygiene practices, the likelihood of poor overall ECD increased as the combined hygiene practice decreased among children with poor hygiene practices (PR_never_: 1.67 [1.40–2.00]; PR_rarely_: 1.49 [1.30–1.71]; PR_sometimes_: 1.30 [1.14–1.49]). Children who did not always follow good hygiene practices had a higher likelihood of poor ECD independently of sociodemographic factors. Considering these findings, future hygiene practice interventions and trials should consider including ECD outcomes.

## 1. Introduction

Promoting hygiene practices is vital for preventing infectious diseases and maintaining good health [1]. Despite the rapid transition and globalisation that many developing countries have experienced in the last three decades, poor sanitation and unhygienic conditions (primarily due to unsafe WAter, lack of Sanitation and lousy Hygiene practices [WASH]) are still of worry. In low- and middle-income countries (LMICs), WASH-related issues are of particular concern due to their direct association with diarrheal death in children under five years [2,3]. In 2020, the Lancet Local Burden of Disease Diarrhoea Collaborators highlighted that the reduction in mortality in some LMICs was directly correlated with the improvements in WASH conditions even though most of the high-risk areas still had poor WASH [3]. Along the same line, oral health conditions continue to rise due to inadequate exposure to fluoride, lack of access to hygiene products, or poor oral hygiene in many LMICs [4]. Yet, the deleterious association of poor sanitation and/or hygiene practices (like poor handwashing and oral hygiene) could potentially extend beyond infections. Previous randomised control trials (RCT) and reviews identified poor hygiene as a risk factor for stunting, iodine deficiency, and inadequate cognitive stimulation and development in children [5,6].

The World Health Organisation (WHO) defines early childhood development (ECD) as ‘the process of cognitive, physical, language, temperament, socioemotional and motor development from conception until age eight’ [7]. This complex construct depends on the interaction of biological, genetic and environmental factors and is the foundation for adulthood well-being and productivity [6]. Unfortunately, around 250 million children under five years are at risk of not achieving their developmental potential [8]. Early learning, responsive caregivers, adequate nutrition and security are the most acknowledged factors to ensure optimal ECD [7]. Secure and safe environments are also vital components that support ECD, as described in the Nurturing Care Framework (NCF) [9].

WASH conditions are particularly important for promoting ECD. The latter is linked with a lower infection rate which has long-term consequences, for instance, for cognitive and language development [10]. However, there are wide disparities in providing suitable WASH conditions globally. For example, in 2015, 39% of the global population had safely managed sanitation services, while—in countries with data available on handwashing—less than 50% of the population had access to basic handwashing facilities [1].

Despite the effect of hygiene and sanitary conditions on infectious disease and child growth, very few studies have investigated their role in ECD [6,11,12]. Previous studies have proposed that infections due to poor hygiene and, consequently, inflammation could connect hygiene practices to child development [6,10,13]. Therefore, considering that the WHO has also highlighted that every child has the right to reach their full potential, identifying and promoting associated factors with development is essential to support optimal development [14]. In this context, this study aimed to investigate the associations of single and combined three hygiene practices with ECD in children in the East Asia and Pacific Region (EAPR).

## 2. Materials and Methods

### 2.1. Participants

This cross-sectional study leveraged data from the East Asia-Pacific Early Child Development Scales (EAP-ECDS). The validation study for the EAP-ECDS was conducted in six countries in East Asia Pacific Region, including Cambodia, China, Mongolia, Papua New Guinea, Timor-Leste, and Vanuatu, to create a standard measurement tool to evaluate the holistic development of children between 3 and 5 years old [15,16]. These countries were selected since they demonstrated the contextual and cultural diversity across the East Asia-Pacific.

The scales were first developed in 2010 and piloted from 2010 to 2012. In those years, the scales were administered to representative samples of children from the countries included to assess their validity and reliability (Cronbach’s alpha was used to assess reliability). Then, the influence of age, gender, and urban-rural residence was considered using country-specific analyses. After that, there was a pilot testing of the scales in 3 countries and subsequent changes to the Scale based on the pilot experience. Finally, the revised 85-item Scale was further validated in the final sample stratified by age, gender and urbanicity in the six aforementioned countries.

After validation, multilevel stratified random sampling was used to select a representative sample from each participating country. The sampling plan was determined in collaboration with the National Census Department or National Statistical Institute in all countries, except in China, where data were collected from five provinces selected to represent varying levels of economic development (Guizhou, Heilongjiang, Jiangsu, Shanghai and Zhejiang) [15,16]. Children with special educational needs, chronic medical conditions (including oedema), and those from ethnic minorities (due to possible differential associations, but the numbers were not sufficient to be analysed separately in the analyses) were excluded from the study. Data collection was carried out between 2012 and 2014, while the analyses for this study were conducted in April 2022 [15,16]. More information about the EAP-ECDS can be found elsewhere [15,16,17,18,19].

### 2.2. Ethical Approval

This study was approved by the Human Research Ethics Committee of The University of Hong Kong. Written informed consent was obtained from the parents of all participants.

### 2.3. Exposure: Hygiene Practices

Trained assessors conducted an interview with parents regarding their child’s health and habits in each country (total administration time was normally 45–60 min). In terms of hygiene practices, caregivers reported information about the following domains: ‘washing hands before a meal,’ ‘washing hands after going to the toilet,’ and ‘brushing teeth.’ Washing hands before a meal and after going to the toilet were initially scored as 0 (never), 1 (rarely), 2 (sometimes), 3 (most of the time) or 4 (always), while brushing teeth as 0 (never), 1 (once), or 2 (twice) a day. These 3 variables were then recoded to have comparable values as (i) ‘always’, children in the highest category in their respective hygiene practice group; (ii) ‘sometimes’, children whom caregivers reported that they rarely, sometimes, or most of the time washed their hands or those who brushed their teeth once per day; and (iii) ‘never’, those in the never category in their respective groups.

To investigate the combined association of these three hygiene practices, a value was assigned to each habit from 0 (never) to 2 (always). An unweighted score was then created as the sum of all 3 items, ranging from 0 (never) to 6 (always). Because of the relatively small number of outcomes in some categories, this total score was reclassified into the following categories: (i) ‘never’ (0 points in the total score), (ii) ‘rarely’ (1 or 2 points in the total score), (iii) ‘sometimes’ (3 or 4 points in the total score), (iv) most of the time to always (5 or 6 points in the total score, hereafter ‘always’).

### 2.4. Outcomes

The EAP-ECDS were administered to children by trained assessors in individual sessions who had experience or training in early childhood education. The assessor administered the test in parallel with the supervisor. In every 20 test administrations, agreement (inter-observer reliability) between the assessor and supervisor was at least 85% before formal testing. The original EAP-ECDS had 85 items that covered seven domains of development: Cognitive Development (21 items); Language and Emergent Literacy Development (henceforth ‘Language Development’; 16 items); Socioemotional Development (15 items); Motor Development (7 items); Health, Hygiene, and Safety Development (9 items); Cultural Knowledge and Participation Development (10 items); and Approaches to Learning Development (7 items) [15]. Overall (total) Development was estimated from the unweighted sum of the domain-specific scores. For this study, overall Development and four specific domains were selected because they are potentially related to hygiene practices: Cognitive Development, Language Development, Socio-emotional Development, and Health, Hygiene and Safety Development. For overall and each specific domain, poor development was identified independently as a binary variable using a threshold score < age-specific 25th centile (in a 1-year band) in the population included in this analysis.

### 2.5. Covariates

Caregivers provided sociodemographic information (sex and area) in individual interviews [15]. Age was calculated using dates of birth and date of assessment. A socioeconomic status (SES) index was created to evaluate multidimensional family SES in this study. The SES index was the first eigenvalue of the principal component analysis results using the correlation matrix from paternal education level, maternal education level, and family assets, including electricity, radio, television, refrigerator, watch, mobile phone, bicycle, animal-drawn cart, agricultural land, livestock, etc. The method has been shown to be valid and reliable for representing the overall SES, as it is shown elsewhere [18,20].

### 2.6. Statistical Analyses

Descriptive characteristics by the individuals and combined hygiene practices are presented as means with standard deviations (SD) for quantitative variables and as frequencies and percentages for categorical variables. Poisson regression models with robust standard errors were used to analyse the cross-sectional associations of the individuals and combined combinations with poor specific-domains development. The results are reported as prevalence ratios (PR) with 95% confidence intervals (CIs) [21]. Poisson regression models with robust standard errors were used because they provide PR estimates which are relatively easy to interpret instead of odds ratios [22]. Robust standard errors were used to correct underinflation when applying the Poisson model for binary outcomes.

All analyses were adjusted for age, sex, country of origin, area, and SES. In addition, a sensitivity analysis was run for combined hygiene practices categories using the total score.

Finally, to investigate whether the associations between ECD and both individual and combined hygiene practice categories differed by subgroups, the analyses were re-run stratified by age category (≥ and < median [4.5 years]), sex (girls and boys), area (urban and rural), SES (≥ and < median), and country of origin (East Asia [China and Mongolia] and Southeast Asia with the Pacific Region [Cambodia, Papua New Guinea, Timor-Leste, and Vanuatu]). These subgroup variables were selected a priori based on their strong influence on child growth and development. Only children with complete data available were included in this study. All statistical analyses were performed using Stata 17.

This study follows the STROBE reporting guidelines for cross-sectional studies [23].

## 3. Results

**Figure 1:**
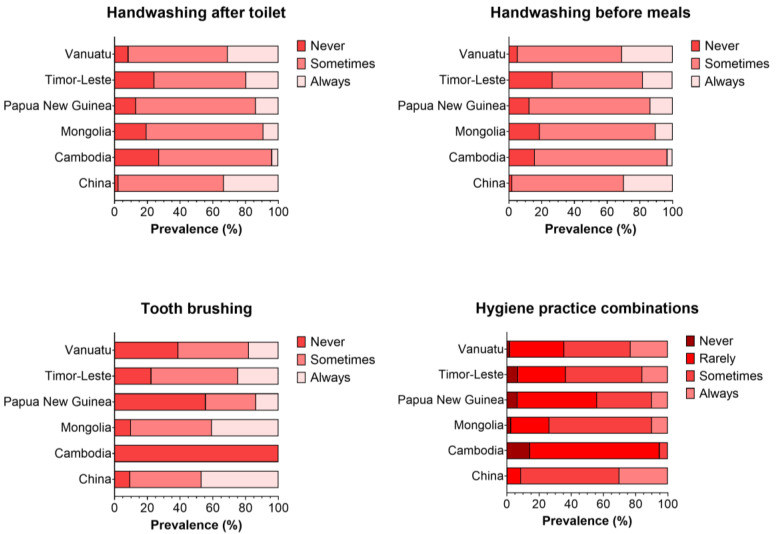
Prevalence of hygiene practice by country of origin. Data presented as prevalence (%) by country of origin by the individual and combined hygiene practices included.

From the original 8296 children included in the EAP-ECDS study, 6697 had complete data available on the exposures (the three hygiene practices included), outcomes (domains included), and covariates (Figure S1). The overall characteristics of the children included by the individual hygiene practice classifications (never, sometimes, always) are shown in Table 1. 13% and 14.2% of the caregivers reported that their children ‘never’ washed their hands before their meals or after going to the toilet, while only 18.2% and 19.1% ‘always’ followed this practice, respectively. In contrast, a higher proportion of children ‘always’ brushed their teeth (27.3%), even though over one-third of children never brushed their teeth. Overall, children who ‘never’ followed any of the individual hygiene practices investigated were younger, more likely to be boys, and tended to come from rural areas and lower SES families (Table 1). Regarding the country of origin, a higher percentage of Chinese children always followed the three hygiene practices investigated (33.3%, 29.8%, and 47% always washed their hands before meals, after going to the toilet, and brushed their teeth, respectively). In contrast, Cambodian children had the highest prevalence of ‘never’ following this practice, especially for the tooth brushing practice (100% declared ‘never’ brushing their teeth; Figure 1). General characteristics by combined hygiene practice classifications are shown in Figure 1 and Figure S2 and Table S1.

Associations between individual hygiene practices and poor domain-specific development are shown in Table 2. Compared with children classified as ‘always’ for washing their hands before a meal, those who washed them ‘sometimes’ (PR: 1.30 [95% CI: 1.16–1.46]) or ‘never’ (PR: 1.35 [1.18–1.55]) had a higher likelihood of poorer Overall ECD. A similar magnitude of association was identified for the cognitive, language, socio-emotional, health, hygiene, and safety development domains (Table 2). Comparable results were observed between the washing hands after going to the toilet hygiene practice and the five development domains investigated. Among them, the domain with the highest likelihood of poor development was Cognitive Development (PR sometimes: 1.40 [1.25–1.58]; PR never: 1.38 [1.19–1.59]). When tooth brushing was used as the exposure, the associations were attenuated and remained only for the never category in all the domains investigated (PR Cognitive Development: 1.30 [1.16–1.46]; PR Overall Development: 1.26 [1.13–1.42]; PR Language Development: 1.26 [1.12–1.42]; PR Health, Hygiene and Safety Development: 1.22 [1.10–1.37]; PR Socioemotional Development: 1.21 [1.08–1.35]; Table 2).

In terms of combined hygiene practice categories, compared with children who always followed all three hygiene practices (washing their hands after going to the toilet and before a meal as well as brushing their teeth), the likelihood of poor Overall ECD increased as the combined hygiene practice decreased, i.e., moving from always to never (see Table 3). For instance, children in the sometimes, rarely and never categories had a 1.30, 1.49, and 1.67-times higher likelihood of poor Overall ECD than their counterparts. Consistent patterns of associations were observed for domain-specific development (Table 3). Associations between ECD domains and the extensive score of the hygiene practice combinations can be found in Table S2.

When moderators stratified the analyses, significant interactions were found for Overall ECD with sex and tooth brushing (*p*-interaction = 0.001); and the three individual domains investigated and area (*p*-interaction < 0.05) as well as the country of origin (*p*-interaction < 0.05; Table 4). Regarding combined hygiene practice categories, a significant interaction was identified for Overall ECD between area and never to sometimes (*p*-interaction < 0.05); and between the country of origin and never to rarely (*p*-interaction < 0.05; Table 5). Other interactions by moderators, individuals and combined hygiene practice categories for the domain-specific outcomes of cognitive, language, socio-emotional, health, hygiene, and safety development can be found in Tables S3 to S10.

## 4. Discussion

Using data from the EAP-ECDS validation study, this study investigated the association between three hygiene practices and the likelihood of poorer ECD in Overall and four domain-specific development. The main findings showed that compared with children who ‘always’ followed any of the individual hygiene practices included, those who ‘sometimes’ or ‘never’ followed whichever of these hygiene practices had a higher likelihood of poor ECD. Among the individual hygiene practices, the highest probability of poor development was observed among children who ‘never’ or ‘sometimes’ washed their hands after going to the toilet (the highest risk was observed for cognitive development). When the three hygiene practices were pooled together to explore the combined associations of hygiene habits, the likelihood of poor development increased as hygiene practices declined. The highest likelihood of poor ECD was observed among children who ‘never’ followed any of the hygiene practices included, with a 67% higher likelihood of poorer overall ECD than their counterparts.

Even though the associations were consistently observed in subgroups, the strength of associations varied by sex, area, and country of origin. These results are not surprising considering that a higher proportion of children who ‘never’ followed some or all hygiene practices were boys and were more likely to come from rural areas. In Cambodia, most of the included children ‘never’ or just ‘sometimes’ followed any hygiene practices, which may be explained by the economic situation of Cambodia, as it remains one of Asia’s poorest countries despite its fast development. This may be associated with poverty and poorly educated mothers in the country. A recent RCT highlighted that children—whose mothers received a trained education on nutrition, oral hygiene and child stimulation during 6 months—had fewer caries (41% vs. 60%, *p* = 0.02), and the use of toothbrushes was more frequent than the control group (66% vs. 38%, *p* = 0.003) [24]. The latter reinforces the family’s role in practical hygiene development, especially mothers. On the other hand, since early childhood education is not compulsory in Cambodia, there is low participation in pre-primary education, mainly linked to rurality [15].

Good hygiene practices (including food preparations) decrease the risk of transmission and ingestion of enteric pathogens, such as a soil-transmitted helminth, which has long-term consequences for cognitive and language development [10]. Moreover, cumulative microbial ingestion may lead to chronic inflammation and can subsequently contribute to malnutrition and altered brain development, two conditions directly associated with ECD during the critical development period [6]. In fact, we previously showed the relevance of nutritional status on ECD using the EAP-ECDS data [25,26]. On the other hand, poor oral health is an important driver of caries, which is associated with speech problems, cognition, and overall development [6,13]. Therefore, infection and inflammation could link hygiene practices to child development [6,13]. In addition to the role in ECD, adherence to WASH practices is associated with a lower risk of COVID-19. This was essential during the last pandemic, where children in Nepal with access to clean water and that self-reported handwashing with soap were associated with a lower risk of this disease, as was highlighted by Shrestha et al. [27].

Poor hygiene practices—combined with inadequate nutrition and lack of stimulation—increase the number of children who are not reaching their full development potential. Notwithstanding the above, few studies have explored the direct association between hygiene practices and ECD. For instance, in a clustered RCT of a nutritional and WASH intervention in Uganda, Muhoozi et al. showed that infants aged 6–8 months in the intervention group had a higher composite development score. The intervention directly impacted their cognitive, language, and motor development [12]. This current study has meaningfully shown that the role of hygiene in ECD could extend well beyond infancy up to at least five years old and can be extended to other LMICs in East Asia and the Pacific region. In contrast, previous RCTs investigated the role of hygiene sanitation interventions on diarrhoea risk (microbes’ burden) and its effect on stunting, which is also a risk factor for poor development [28,29].

Optimal ECD might be achievable with low-cost strategies [30]. Environments that could facilitate better hygiene practices, as well as educational interventions, were shown to improve WASH behaviours and subsequently reduce infectious diseases. Following the hypothesis that WASH behaviours could improve ECD by reducing infectious disease, these interventions should also impact ECD [31]. However, despite its relevance for each individual and society, along with the numerous programmes and implementations carried out in LMICs, barriers against ECD persist [32]. Among them, the latest Estimating Global Water, Sanitation, and Hygiene Levels and Related Risks on Human Health Report (1990–2020) [33] underlined how poverty in LMICs still affects access to clean water. Additionally, the report highlighted that adults had a higher likelihood of access to clean water than children and the elderly. Hence, caregivers are role models for children and, therefore, must be the first to follow good hygiene practices. Yet, a recent review identified that a lack of knowledge of critical times for handwashing, a lack of importance of washing some food before eating and a lack of resources (such as water and soap) were part of the boundaries among caregivers associated with poorer children hygiene practices [34]. In this context, and in terms of unhygienic environments, WASH interventions need to remain in place whilst also addressing important vectors such as soil, faecal contamination, and infant food, as these may be pathways for microbes’ ingestion, which is linked to poor ECD [6]. In doing so, and in line with the Sustainable Development Goals, better hygiene practices (including handwashing facilities with soap and water) should be achievable for every child by 2030 [14].

### Strengths and Limitations

This study leveraged data from the EAP-ECDS validation study and was a population-representative survey conducted in six countries in the EAPR [15]. In addition, the EAP-ECDS domains were objectively measured by professionals with training in early childhood education, which increases the confidence in measurement accuracy. Nonetheless, this study is not without limitations. Firstly, this study only included three hygiene practices that the caregiver of each child reported. Other key hygiene factors, such as access to clean water and sanitation, caregiver hygiene practices, as well as food hygiene preparation, were not measured. This could have enhanced our understanding of the overview of hygiene environments by country beyond individual hygiene practices. The hygiene practices were not observed objectively and could be biased. Moreover, it is unclear if the handwashing practice after going to the toilet or before a meal was carried out with soap. Secondly, other confounding factors such as physical activity, adequate nutrition and responsive caregiving were not reported. These factors are critical components of naturing care related to ECD [7,9,35]. The associations specific to the country also could not be studied due to the smaller sample size. Finally, due to the cross-sectional nature of this study, causality cannot be inferred.

## 5. Conclusions

Children who did not always follow good hygiene practices had a higher likelihood of poor development independently of sociodemographic factors. Environments that could facilitate better hygiene practices have been shown to improve WASH behaviours and reduce infectious diseases. Yet, this goal is still not achievable in LMICs, where poverty continues to be the main barrier. This study re-emphasises the importance of WASH behaviours in ECD. Considering these findings, future studies and interventions should include ECD outcomes. 

## Figures and Tables

**Table 1:** General characteristics by individual hygiene practice.

	Overall	Wash Hands before a Meal			Wash Hands after Going to the Toilet			Tooth Brushing		
		Never	Sometimes	Always	Never	Sometimes	Always	Never	Sometimes	Always
**Total, *n* (%)**	6697 (100)	867 (13.0)	4608 (68.8)	1222 (18.2)	953 (14.2)	4462 (66.7)	1282 (19.1)	2259 (33.7)	2612 (39.0)	1286 (27.3)
Age (years), mean (SD)	4.0 (0.8)	3.9 (0.8)	4.0 (0.8)	4.2 (0.8)	3.8 (0.8)	4.0 (0.8)	4.2 (0.8)	3.9 (0.9)	4.0 (0.8)	4.1 (0.8)
Sex, *n* (%)										
Girls	3337 (49.8)	426 (49.1)	2287 (49.6)	624 (51.1)	452 (47.4)	2223 (49.8)	662 (51.6)	1074 (47.5)	1303 (49.9)	960 (52.6)
Boys	3360 (50.2)	441 (50.9)	2321 (50.4)	598 (48.9)	501 (52.6)	2239 (50.2)	620 (48.4)	1185 (52.5)	1309 (50.1)	866 (47.4)
Area, *n* (%)										
Rural	3999 (59.7)	613 (70.7)	2719 (59.0)	667 (54.6)	679 (71.2)	2646 (59.3)	674 (52.6)	1704 (75.4)	1508 (57.7)	787 (43.1)
Urban	2698 (40.3)	254 (29.3)	1889 (41.0)	555 (45.4)	274 (28.8)	1816 (40.7)	608 (47.4)	555 (24.6)	1104 (42.3)	1039 (56.9)
SES, mean (SD)	0.04 (1.5)	−0.39 (1.6)	0.06 (1.5)	0.25 (1.4)	−0.45 (1.6)	0.07 (1.5)	0.30 (1.4)	−0.82 (1.3)	0.21 (1.5)	0.86 (1.3)

Data are presented as means with SD for quantitative variables and as frequencies and percentages for categorical variables. SES: composite socioeconomic z-score; SD: standard deviations.

**Table 2:** Individual hygiene habits and their association with ECD in children.

	Never		Sometimes		Always
	PR (95% CI)	*p*-Value	PR (95% CI)	*p*-Value	PR (95% CI)
**Wash hands before a meal**					
Total development	1.35 (1.18; 1.55)	<0.001	1.30 (1.16; 1.46)	<0.001	1.00 (Ref.)
Cognitive development	1.18 (1.02; 1.38)	0.030	1.38 (1.22; 1.55)	<0.001	1.00 (Ref.)
Language development	1.28 (1.11; 1.48)	0.001	1.32 (1.18; 1.49)	<0.001	1.00 (Ref.)
Socioemotional development	1.27 (1.11; 1.45)	<0.001	1.20 (1.07; 1.34)	0.001	1.00 (Ref.)
Health hygiene and safety development	1.16 (1.01; 1.33)	0.032	1.23 (1.10; 1.36)	<0.001	1.00 (Ref.)
**Wash hands after going to the toilet**					
Total development	1.34 (1.18; 1.53)	<0.001	1.21 (1.08; 1.35)	0.001	1.00 (Ref.)
Cognitive development	1.38 (1.19; 1.59)	<0.001	1.40 (1.25; 1.58)	<0.001	1.00 (Ref.)
Language development	1.24 (1.09; 1.42)	0.001	1.16 (1.04; 1.29)	0.009	1.00 (Ref.)
Socioemotional development	1.33 (1.17; 1.52)	<0.001	1.21 (1.08; 1.35)	0.001	1.00 (Ref.)
Health hygiene and safety development	1.32 (1.16; 1.50)	<0.001	1.27 (1.14; 1.41)	<0.001	1.00 (Ref.)
**Tooth brushing**					
Total development	1.26 (1.13; 1.42)	<0.001	0.94 (0.84; 1.05)	0.295	1.00 (Ref.)
Cognitive development	1.30 (1.16; 1.46)	<0.001	1.08 (0.96; 1.21)	0.180	1.00 (Ref.)
Language development	1.26 (1.12; 1.42)	<0.001	0.95 (0.84; 1.07)	0.371	1.00 (Ref.)
Socioemotional development	1.21 (1.08; 1.35)	0.001	0.94 (0.84; 1.05)	0.264	1.00 (Ref.)
Health hygiene and safety development	1.22 (1.10; 1.37)	<0.001	0.99 (0.89; 1.11)	0.925	1.00 (Ref.)

Data presented as prevalence ratio (PR) and 95% CIs for poor development (score < 25th centile-age). Children who always washed their hands before meals, who always washed their hands after going to the bathroom, or those who always brushed their teeth were used as the reference group per each case. For tooth brushing, sometimes means once a day. Analyses were adjusted by age, sex, SES, area, and country of origin.

**Table 3:** Combinations of hygiene habits and their association with ECD in children (re-classification).

	Never		Rarely		Sometimes		Always
	PR (95% CI)	*p*-Value	PR (95% CI)	*p*-Value	PR (95% CI)	*p*-Value	PR (95% CI)
Overall Development	1.67 (1.40; 2.00)	<0.001	1.49 (1.30; 1.71)	<0.001	1.30 (1.14; 1.49)	<0.001	1.00 (Ref.)
Cognitive Development	1.58 (1.29; 1.94)	<0.001	1.61 (1.39; 1.87)	<0.001	1.51 (1.30.; 1.75)	<0.001	1.00 (Ref.)
Language Development	1.57 (1.33; 1.85)	<0.001	1.33 (1.16; 1.52)	<0.001	1.16 (1.01; 1.33)	0.036	1.00 (Ref.)
Socioemotional Development	1.58 (1.33; 1.88)	<0.001	1.37 (1.20; 1.57)	<0.001	1.23 (1.08; 1.41)	0.002	1.00 (Ref.)
Health, Hygiene and Safety Development	1.48 (1.24; 1.77)	<0.001	1.38 (1.21; 1.58)	<0.001	1.28 (1.13; 1.46)	<0.001	1.00 (Ref.)

Data presented as prevalence ratio (PR) and 95% CIs for poor development (score <25th centile-age). Children who always washed their hands before meals, after going to the bathroom and brushed their teeth, were used as the reference group. Analyses were adjusted by age, sex, SES, area and country of origin.

**Table 4:** Individual hygiene practices and their association with Overall Development in children by moderators.

	Wash Hands before a Meal			Wash Hands after Going to the Toilet			Tooth Brushing		
	Never	Sometimes	Always	Never	Sometimes	Always	Never	Sometimes	Always
	PR (95% CI)	PR (95% CI)	PR (95% CI)	PR (95% CI)	PR (95% CI)	PR (95% CI)	PR (95% CI)	PR (95% CI)	PR (95% CI)
**Age**									
<median	1.42 ** (1.15; 1.74)	1.28 ** (1.07; 1.52)	1.00 (Ref.)	1.34 ** (1.10; 1.63)	1.13 (0.96; 1.34)	1.00 (Ref.)	1.26 ** (1.07; 1.49)	0.90 (0.77; 1.07)	1.00 (Ref.)
≥median	1.31 ** (1.09; 1.58)	1.32 *** (1.14; 1.54)	1.00 (Ref.)	1.36 *** (1.14; 1.61)	1.28 ** (1.11; 1.47)	1.00 (Ref.)	1.27 ** (1.10; 1.47)	0.97 (0.83; 1.12)	1.00 (Ref.)
P_interaction_	0.403	0.972		0.980	0.453		0.068	0.219	
**Sex**									
Female	1.32 ** (1.09; 1.61)	1.34 *** (1.14; 1.58)	1.00 (Ref.)	1.32 ** (1.10; 1.59)	1.22 * (1.04; 1.42)	1.00 (Ref.)	1.46 *** (1.25; 1.71)	1.04 (0.89; 1.22)	1.00 (Ref.)
Male	1.37 ** (1.13; 1.66)	1.27 ** (1.08; 1.49)	1.00 (Ref.)	1.35 ** (1.12; 1.62)	1.19 * (1.02; 1.39)	1.00 (Ref.)	1.10 (0.94; 1.29)	0.86 (0.74; 1.00)	1.00 (Ref.)
P_interaction_	0.892	0.489		0.945	0.767		0.001	0.070	
**Area**									
Rural	1.56 *** (1.27; 1.92)	1.57 *** (1.31; 1.88)	1.00 (Ref.)	1.62 *** (1.33; 1.97)	1.43 *** (1.20; 1.71)	1.00 (Ref.)	1.55 *** (1.29; 1.86)	1.07 (0.88; 1.30)	1.00 (Ref.)
Urban	1.24 * (1.02; 1.50)	1.07 (0.92; 1.24)	1.00 (Ref.)	1.12 (0.93; 1.36)	1.03 (0.90; 1.19)	1.00 (Ref.)	1.02 (0.87; 1.18)	0.91 (0.79; 1.04)	1.00 (Ref.)
P_interaction_	0.009	<0.001		<0.001	0.002		0.003	0.210	
**SES**									
<median	1.35 *** (1.16; 1.58)	1.34 *** (1.17; 1.53)	1.00 (Ref.)	1.41 *** (1.22; 1.64)	1.25 ** (1.10; 1.42)	1.00 (Ref.)	1.32 *** (1.16; 1.51)	0.94 (0.82; 1.08)	1.00 (Ref.)
≥median	1.42 * (1.07; 1.89)	1.18 (0.94; 1.48)	1.00 (Ref.)	1.22 (0.92; 1.62)	1.12 (0.91; 1.38)	1.00 (Ref.)	1.12 (0.89; 1.42)	0.97 (0.81; 1.16)	1.00 (Ref.)
P_interaction_	0.949	0.237		0.182	0.267		0.205	0.664	
**Country of origin**									
China-Mongolia	5.89 *** (3.10; 11.2)	1.85 * (1.01; 3.39)	1.00 (Ref.)	5.96 *** (3.04; 11.7)	2.28 * (1.21; 4.29)	1.00 (Ref.)	1.52 (0.90; 2.56)	1.10 (0.75; 1.63)	1.00 (Ref.)
Others	1.37 *** (1.20; 1.58)	1.27 *** (1.13; 1.43)	1.00 (Ref.)	1.31 *** (1.15; 1.49)	1.18 ** (1.06; 1.32)	1.00 (Ref.)	1.04 (0.93; 1.16)	0.93 (0.83; 1.04)	1.00 (Ref.)
P_interaction_	<0.001	0.164		<0.001	0.027		0.015	0.106	

Data presented as prevalence ratio (PR) and 95% CIs for poor development (score < 25th centile-age). Children who always washed their hands before meals, who always washed their hands after going to the bathroom, or those who always brushed their teeth were used as the reference group per each case. For tooth brushing, sometimes means once a day. Analyses were adjusted by age, sex, SES, area, and country of origin when these were not the moderators. Significance was denoted as *** (*p* < 0.001), ** (*p* < 0.01), * (*p* < 0.05).

**Table 5:** Combinations of hygiene practices and their association with Overall Development in children by moderators.

	Never	Rarely	Sometimes	Always
	PR (95% CI)	PR (95% CI)	PR (95% CI)	PR (95% CI)
**Age**				
<median	1.83 (1.41; 2.37) ***	1.44 (1.16; 1.79) **	1.26 (1.02; 1.57) *	1.00 (Ref.)
≥median	1.56 (1.22; 1.99) ***	1.53 (1.28; 1.82) ***	1.34 (1.12; 1.60) **	1.00 (Ref.)
P_interaction_	0.541	0.440	0.972	
**Sex**				
Female	1.69 (1.30; 2.20) ***	1.64 (1.34; 2.00) ***	1.39 (1.14; 1.70) **	1.00 (Ref.)
Male	1.64 (1.29; 2.08) ***	1.34 (1.11; 1.62) **	1.22 (1.01; 1.47) *	1.00 (Ref.)
P_interaction_	0.666	0.076	0.389	
**Area**				
Rural	2.14 (1.63; 2.80) ***	1.99 (1.57; 2.52) ***	1.59 (1.25; 2.03) ***	1.00 (Ref.)
Urban	1.41 (1.06; 1.87) *	1.12 (0.94; 1.34)	1.12 (0.95; 1.32)	1.00 (Ref.)
P_interaction_	0.007	<0.001	0.010	
**SES**				
<median	1.77 (1.45; 2.16) ***	1.55 (1.32; 1.83) ***	1.32 (1.12; 1.55) **	1.00 (Ref.)
≥median	1.43 (0.88; 2.32)	1.37 (1.04; 1.79) *	1.27 (0.99; 1.62)	1.00 (Ref.)
P_interaction_	0.321	0.250	0.686	
**Country of origin**				
East Asia	10.0 (4.00; 25.2) ***	4.45 (2.23; 8.86) ***	2.17 (1.12; 4.20) *	1.00 (Ref.)
Southeast Asia and Pacific Region	1.52 (1.27; 1.81) ***	1.32 (1.15; 1.52) ***	1.32 (1.15; 1.52) ***	1.00 (Ref.)
P_interaction_	<0.001	<0.001	0.104	

Data presented as prevalence ratio (PR) and 95% CIs for poor development (score <25th centile-age). Children who always washed their hands before meals, after going to the bathroom and brushed their teeth, were used as the reference group. Analyses were adjusted by age, sex, SES, area, and country of origin when these were not the moderators. East Asia: China and Mongolia, Southeast Asia and Pacific region: Cambodia, Timor-Leste, Papua New Guinea, Vanuatu. Significance was denoted as *** (*p* < 0.001), ** (*p* < 0.01), * (*p* < 0.05).

## Data Availability

Data are available via NR upon reasonable request.

## Supplementary Materials

The following supporting information can be downloaded at: https://www.mdpi.com/article/10.3390/ijerph20042798/s1, Table S1. Characteristics by combined categories; Table S2. Combinations of hygiene practices and their association with ECD in children; Table S3. Individual hygiene practices and their association with Cognitive Development in children by moderators; Table S4. Individual hygiene practices and their association with Language Development in children by moderators; Table S5. Individual hygiene practices and their association with Socio-emotional Development in children by moderators; Table S6. Individual hygiene practices and their association with Health, Hygiene and Safety Development in children by moderators; Table S7. Combinations of hygiene practices and their association with Cognitive Development in children by moderators; Table S8. Combinations of hygiene practices and their association with Language Development in children by moderators; Table S9. Combinations of hygiene practices and their association with Socio-emotional Development in children by moderators; Table S10. Combinations of hygiene practices and their association with Health, Hygiene and Safety Development in children by moderators; Figure S1. Diagram children included in the study; Figure S2. Prevalence of combined hygiene practice by country of origin.
