# Supplementary material for: Hygiene Practices and Early Childhood Development in the East Asia-Pacific Region: A Cross-Sectional Analysis
Source: Int J Environ Res Public Health. 2023 Feb 4;20(4):2798. doi: 10.3390/ijerph20042798 (PMC9957383; doi:10.3390/ijerph20042798)
Supplement: Supplementary file 1 [file ijerph-20-02798-s001.zip › ijerph-2065757-supplementary.pdf]

Supplementary Material

Hygiene Practices and Early Childhood Development in the East Asia-Pacific Region: a cross-sectional analysis

Table S1. Characteristics by combined categories. 2

Table S2. Combinations of hygiene practices and their association with ECD in children 3

Table S3. Individual hygiene practices and their association with Cognitive Development in children by moderators 4

Table S4. Individual hygiene practices and their association with Language Development in children by moderators 5

Table S5. Individual hygiene practices and their association with Socio-emotional Development in children by moderators 6

Table S6. Individual hygiene practices and their association with Health, Hygiene and Safety Development in children by moderators 7

Table S7. Combinations of hygiene practices and their association with Cognitive Development in children by moderators 8

Table S8. Combinations of hygiene practices and their association with Language Development in children by moderators 9

Table S9. Combinations of hygiene practices and their association with Socio-emotional Development in children by moderators 10

Table S10. Combinations of hygiene practices and their association with Health, Hygiene and Safety Development in children by moderators 11

Figure S1. Diagram children included in the study. 12

Figure S2. Prevalence of combined hygiene practice by country of origin 13

Table S1. Characteristics by combined categories.

	Overall	Combines hygiene practice categories			
		**Never**	**Rarely**	**Sometimes**	**Always**
Total, n (%)	6697 (100)	319	2229	3088	1061
Age (years), mean (SD)	4·0 (0·8)	3.7 (0.8)	4.0 (0.8)	4.0 (0.8)	4.2 (0.8)
Sex, n (%)					
Girls	3337 (49.8)	152 (47.6)	1064 (47.7)	1564 (50.6)	557 (52.5)
Boys	3360 (50.2)	167 (52.4)	1165 (52.3)	1524 (49.4)	504 (47.5)
Area, n (%)					
Rural	3999 (59.7)	263 (82.4)	1612 (72.3)	1588 (51.4)	536 (50.5)
Urban	2698 (40.3)	56 (17.6)	617 (27.7)	1500 (48.6)	525 (49.5)
SES, mean (SD)	0.04 (1.5)	-1.1 (1.4)	-0.61 (1.4)	0.48 (1.4)	0.45 (1.4)

Data presented as means with SD for quantitative variables and as frequencies and percentages for categorical variables. SES: composite socioeconomic z-score; SD: standard deviations.

Table S2. Combinations of hygiene practices and their association with ECD in children

	0		1		2		3		4		5		6
	PR (95% CI)	p-value	PR (95% CI)	p-value	PR (95% CI)	p-value	PR (95% CI)	p-value	PR (95% CI)	p-value	PR (95% CI)	p-value	PR (95% CI)
Overall Development	1.55 (1.22; 1.97)	<0.001	1.48 (1.18; 1.86)	0.001	1.36 (1.10; 1.68)	0.005	1.17 (0.94; 1.45)	0.153	1.29 (1.04; 1.61)	0.022	0.90 (0.70; 1.16)	0.416	1.00 (Ref.)
Cognitive Development	1.44 (1.11; 1.86)	0.006	1.40 (1.09; 1.80)	0.008	1.48 (1.19; 1.85)	<0.001	1.38 (1.10; 1.72)	0.005	1.35 (1.08; 1.70)	0.009	0.85 (0.65; 1.12)	0.259	1.00 (Ref.)
Language Development	1.69 (1.32; 2.17)	<0.001	1.41 (1.10; 1.81)	0.006	1.44 (1.14; 1.81)	0.002	1.21 (0.96; 1.53)	0.108	1.33 (1.04; 1.68)	0.021	1.13 (0.87; 1.47)	0.367	1.00 (Ref.)
Socioemotional Development	1.59 (1.25; 2.01)	<0.001	1.46 (1.16; 1.84)	0.001	1.35 (1.09; 1.67)	0.006	1.19 (0.96; 1.48)	0.107	1.32 (1.06; 1.64)	0.015	1.01 (0.79; 1.30)	0.919	1.00 (Ref.)
Health, Hygiene and Safety Development	1.60 (1.25; 2.04)	<0.001	1.49 (1.17; 1.89)	0.001	1.48 (1.19; 1.84)	<0.001	1.36 (1.09; 1.69)	0.006	1.42 (1.13; 1.78)	0.002	1.12 (0.87; 1.44)	0.377	1.00 (Ref.)

Data presented as prevalence ratio (PR) and 95% CIs for poor development (score <25th centile-age). Children who always washed their hands before meals, after going to the bathroom and brushing their teeth, were used as the reference group. Analyses were adjusted by age, sex, SES, area, and country of origin.

Table S3. Individual hygiene practices and their association with Cognitive Development in children by moderators

	Wash hands before a meal			Wash hands after going to the toilet			Tooth brushing		
	**Never**	**Sometimes**	**Always**	**Never**	**Sometimes**	**Always**	**Never**	**Sometimes**	**Always**
	**PR (95% CI)**	**PR (95% CI)**	**PR (95% CI)**	**PR (95% CI)**	**PR (95% CI)**	**PR (95% CI)**	**PR (95% CI)O**	**PR (95% CI)**	**PR (95% CI)**
**Age**									
< median	1.22 (0.96; 1.54)	1.42 (1.17; 1.71)***	1.00 (Ref.)	1.45 (1.15; 1.83)**	1.46 (1.20; 1.78)***	1.00 (Ref.)	1.35 (1.13; 1.61)**	1.16 (0.98; 1.37)	1.00 (Ref.)
≥ median	1.19 (0.97; 1.46)	1.34 (1.15; 1.55)***	1.00 (Ref.)	1.34 (1.11; 1.62)**	1.37 (1.18; 1.58)***	1.00 (Ref.)	1.26 (1.08; 1.46)**	0.98 (0.84; 1.14)	1.00 (Ref.)
$P_{interaction}$	0.474	0.336		0.351	0.314		0.057	0.837	
**Sex**									
Female	1.26 (1.02; 1.55)*	1.39 (1.18; 1.64)***	1.00 (Ref.)	1.36 (1.11; 1.65)**	1.32 (1.12; 1.54)**	1.00 (Ref.)	1.28 (1.10; 1.50)**	1.07 (0.92; 1.25)	1.00 (Ref.)
Male	1.12 (0.89; 1.40)	1.36 (1.15; 1.62)***	1.00 (Ref.)	1.41 (1.13; 1.75)**	1.51 (1.26; 1.81)***	1.00 (Ref.)	1.31 (1.10; 1.56)**	1.09 (0.92; 1.29)	1.00 (Ref.)
$P_{interaction}$	0.422	0.721		0.924	0.328		0.298	0.926	
**Area**									
Rural	1.36 (1.09; 1.69)**	1.66 (1.39; 1.99)***	1.00 (Ref.)	1.66 (1.34; 2.06)***	1.69 (1.40; 2.04)***	1.00 (Ref.)	1.63 (1.35; 1.97)***	1.23 (1.01; 1.49)*	1.00 (Ref.)
Urban	1.12 (0.90; 1.41)	1.11 (0.95; 1.29)	1.00 (Ref.)	1.20 (0.96; 1.49)	1.16 (0.99; 1.35)	1.00 (Ref.)	1.00 (0.86; 1.17)	1.02 (0.88; 1.17)	1.00 (Ref.)
$P_{interaction}$	0.052	0.001		0.003	0.002		0.035	0.383	
**SES**									
< median	1.23 (1.04; 1.47)*	1.41 (1.23; 1.62)***	1.00 (Ref.)	1.43 (1.21; 1.69)***	1.42 (1.24; 1.63)***	1.00 (Ref.)	1.35 (1.18; 1.55)***	1.01 (0.88; 1.17)	1.00 (Ref.)
≥ median	1.12 (0.82; 1.53)	1.26 (0.99; 1.58)	1.00 (Ref.)	1.34 (0.98; 1.81)	1.38 (1.09; 1.73)**	1.00 (Ref.)	1.05 (0.83; 1.34)	1.16 (0.97; 1.39)	1.00 (Ref.)
$P_{interaction}$	0.811	0.510		0.905	0.971		0.772	0.062	
**Country of origin**									
East Asia	3.06 (1.96; 4.79)***	1.80 (1.24; 2.63)**	1.00 (Ref.)	3.75 (2.36; 5.97)***	2.27 (1.52; 3.40)***	1.00 (Ref.)	1.64 (1.15; 2.34)**	1.35 (1.04; 1.75)*	1.00 (Ref.)
Southeast Asia and Pacific Region	1.11 (0.94; 1.30)	1.37 (1.21; 1.55)***	1.00 (Ref.)	1.27 (1.09; 1.48)**	1.41 (1.24; 1.59)***	1.00 (Ref.)	1.17 (1.04; 1.31)*	0.99 (0.88; 1.13)	1.00 (Ref.)
$P_{interaction}$	<0.001	0.076		<0.001	0.007		<0.001	0.001	

Data presented as prevalence ratio (PR) and 95% CIs for poor development (score <25th centile-age). Children who always washed their hands before meals, or who always washed their hands after going to the bathroom, or those who always brushing their teeth were used as the reference group per each case. For tooth brushing, sometimes means once a day. Analyses were adjusted by age, sex, SES, area, and country of origin when these were not the moderators.

East Asia: China and Mongolia, Southeast Asia and Pacific region: Cambodia, Timor-Leste, Papua New Guinea, Vanuatu. Significance was denoted as *** (p<0.001), ** (p<0.01), * (p<0.05).

Table S4. Individual hygiene practices and their association with Language Development in children by moderators

	Wash hands before a meal			Wash hands after going to the toilet			Tooth brushing		
	Never	Sometimes	Always	Never	Sometimes	Always	Never	Sometimes	Always
	PR (95% CI)	PR (95% CI)	PR (95% CI)	PR (95% CI)	PR (95% CI)	PR (95% CI)	PR (95% CI)	PR (95% CI)	PR (95% CI)
**Age**									
< median	1.29 (1.04; 1.60)*	1.29 (1.07; 1.55)**	1.00 (Ref.)	1.21 (0.98; 1.49)	1.11 (0.93; 1.33)	1.00 (Ref.)	1.21 (1.00; 1.45)*	0.84 (0.70; 1.01)	1.00 (Ref.)
≥ median	1.30 (1.08; 1.57)**	1.36 (1.17; 1.58)***	1.00 (Ref.)	1.31 (1.11; 1.55)**	1.22 (1.06; 1.40)**	1.00 (Ref.)	1.34 (1.15; 1.55)***	1.03 (0.85; 1.20)	1.00 (Ref.)
$P_{interaction}$	0.601	0.914		0.887	0.842		0.449	0.066	
**Sex**									
Female	1.27 (1.03; 1.55)*	1.32 (1.11; 1.57)**	1.00 (Ref.)	1.29 (1.07; 1.56)**	1.18 (1.01; 1.38)*	1.00 (Ref.)	1.34 (1.14; 1.59)***	0.98 (0.82; 1.16)	1.00 (Ref.)
Male	1.30 (1.06; 1.58)*	1.32 (1.13; 1.56)**	1.00 (Ref.)	1.20 (0.99; 1.43)	1.13 (0.97; 1.31)	1.00 (Ref.)	1.17 (0.99; 1.39)	0.92 (0.77; 1.08)	1.00 (Ref.)
$P_{interaction}$	0.637	0.669		0.212	0.389		0.050	0.457	
**Area**									
Rural	1.34 (1.11; 1.62)**	1.41 (1.20; 1.66)***	1.00 (Ref.)	1.43 (1.20; 1.71)***	1.30 (1.11; 1.53)**	1.00 (Ref.)	1.50 (1.24; 1.81)***	1.06 (0.87; 1.29)	1.00 (Ref.)
Urban	1.26 (1.01; 1.59)*	1.22 (1.03; 1.44)*	1.00 (Ref.)	1.06 (0.85; 1.32)	1.02 (0.88; 1.19)	1.00 (Ref.)	1.06 (0.90; 1.26)	0.92 (0.79; 1.07)	1.00 (Ref.)
$P_{interaction}$	0.225	0.077		0.001	0.008		0.002	0.362	
**SES**									
< median	1.33 (1.15; 1.55)***	1.31 (1.15; 1.49)***	1.00 (Ref.)	1.30 (1.13; 1.49)***	1.12 (0.99; 1.27)	1.00 (Ref.)	1.35 (1.18; 1.55)***	0.98 (0.85; 1.13)	1.00 (Ref.)
≥ median	1.12 (0.76; 1.65)	1.36 (1.04; 1.77)*	1.00 (Ref.)	1.01 (0.68; 1.49)	1.27 (0.99; 1.63)	1.00 (Ref.)	1.06 (0.80; 1.41)	0.93 (0.74; 1.16)	1.00 (Ref.)
$P_{interaction}$	0.232	0.983		0.079	0.495		0.037	0.898	
**Country of origin**									
East Asia	6.04 (1.93; 18.9)**	2.99 (1.07; 8.34)*	1.00 (Ref.)	5.22 (1.63; 16.7)**	3.36 (1.19; 9.51)*	1.00 (Ref.)	2.27 (1.10; 4.65)*	0.99 (0.54; 1.80)	1.00 (Ref.)
Southeast Asia and Pacific Region	1.32 (1.15; 1.52)***	1.34 (1.19; 1.51)***	1.00 (Ref.)	1.28 (1.12; 1.45)***	1.17 (1.05; 1.31)**	1.00 (Ref.)	1.19 (1.06; 1.33)**	0.94 (0.83; 1.06)	1.00 (Ref.)
$P_{interaction}$	0.005	0.106		0.007	0.036		0.011	0.527	

Data presented as prevalence ratio (PR) and 95% CIs for poor development (score <25th centile-age). Children who always washed their hands before meals, or who always washed their hands after going to the bathroom, or those who always brushing their teeth were used as the reference group per each case. For tooth brushing, sometimes means once a day. Analyses were adjusted by age, sex, SES, area, and country of origin when these were not the moderators.

East Asia: China and Mongolia, Southeast Asia and Pacific region: Cambodia, Timor-Leste, Papua New Guinea, Vanuatu. Significance was denoted as *** (p<0.001), ** (p<0.01), * (p<0.05).

Table S5. Individual hygiene practices and their association with Socio-emotional Development in children by moderators

	Wash hands before a meal			Wash hands after going to the toilet			Tooth brushing		
	Never	Sometimes	Always	Never	Sometimes	Always	Never	Sometimes	Always
	PR (95% CI)	PR (95% CI)	PR (95% CI)	PR (95% CI)	PR (95% CI)	PR (95% CI)	PR (95% CI)	PR (95% CI)	PR (95% CI)
**Age**									
< median	1.29 (1.06; 1.56)*	1.15 (0.98; 1.36)	1.00 (Ref.)	1.33 (1.10; 1.61)**	1.13 (0.95; 1.33)	1.00 (Ref.)	1.10 (0.94; 1.29)	0.87 (0.75; 1.02)	1.00 (Ref.)
≥ median	1.26 (1.05; 1.51)*	1.23 (1.06; 1.43)**	1.00 (Ref.)	1.32 (1.11; 1.57)**	1.27 (1.10; 1.47)**	1.00 (Ref.)	1.30 (1.12; 1.51)***	0.99 (0.85; 1.15)	1.00 (Ref.)
$P_{interaction}$	0.798	0.666		0.999	0.372		0.001	0.077	
**Sex**									
Female	1.35 (1.11; 1.63)**	1.28 (1.09; 1.51)**	1.00 (Ref.)	1.45 (1.20; 1.76)***	1.28 (1.09; 1.51)**	1.00 (Ref.)	1.25 (1.07; 1.45)**	0.93 (0.80; 1.08)	1.00 (Ref.)
Male	1.20 (0.99; 1.45)	1.12 (0.96; 1.31)	1.00 (Ref.)	1.21 (1.01; 1.46)*	1.13 (0.97; 1.31)	1.00 (Ref.)	1.16 (0.99; 1.37)	0.95 (0.81; 1.11)	1.00 (Ref.)
$P_{interaction}$	0.197	0.138		0.084	0.161		0.101	0.837	
**Area**									
Rural	1.37 (1.13; 1.66)**	1.36 (1.15; 1.61)***	1.00 (Ref.)	1.59 (1.31; 1.92)***	1.43 (1.20; 1.70)***	1.00 (Ref.)	1.37 (1.16; 1.63)***	1.01 (0.84; 1.21)	1.00 (Ref.)
Urban	1.23 (1.02; 1.49)*	1.04 (0.90; 1.22)	1.00 (Ref.)	1.12 (0.92; 1.36)	1.04 (0.90; 1.20)	1.00 (Ref.)	1.07 (0.91; 1.26)	0.92 (0.80; 1.06)	1.00 (Ref.)
$P_{interaction}$	0.212	0.005		0.002	0.001		0.029	0.642	
**SES**									
< median	1.25 (1.08; 1.46)**	1.22 (1.08; 1.39)**	1.00 (Ref.)	1.34 (1.16; 1.56)***	1.24 (1.09; 1.40)**	1.00 (Ref.)	1.28 (1.12; 1.46)***	0.94 (0.82; 1.08)	1.00 (Ref.)
≥ median	1.39 (1.06; 1.82)*	1.09 (0.87; 1.36)	1.00 (Ref.)	1.40 (1.07; 1.84)*	1.10 (0.89; 1.37)	1.00 (Ref.)	1.01 (0.79; 1.29)	0.96 (0.81; 1.15)	1.00 (Ref.)
$P_{interaction}$	0.415	0.366		0.630	0.411		0.148	0.599	
**Country of origin**									
East Asia	4.42 (2.71; 7.23)***	1.71 (1.09; 2.69)*	1.00 (Ref.)	5.05 (3.00; 8.52)***	2.22 (1.36; 3.62)**	1.00 (Ref.)	1.22 (0.78; 1.91)	1.14 (0.84; 1.55)	1.00 (Ref.)
Southeast Asia and Pacific Region	1.32 (1.16; 1.51)***	1.21 (1.08; 1.35)**	1.00 (Ref.)	1.33 (1.17; 1.52)***	1.21 (1.08; 1.35)**	1.00 (Ref.)	1.04 (0.94; 1.16)	0.91 (0.81; 1.02)	1.00 (Ref.)
$P_{interaction}$	<0.001	0.085		<0.001	0.008		0.062	0.018	

Data presented as prevalence ratio (PR) and 95% CIs for poor development (score <25th centile-age). Children who always washed their hands before meals, or who always washed their hands after going to the bathroom, or those who always brushing their teeth were used as the reference group per each case. For tooth brushing, sometimes means once a day. Analyses were adjusted by age, sex, SES, area, and country of origin when these were not the moderators.

East Asia: China and Mongolia, Southeast Asia and Pacific region: Cambodia, Timor-Leste, Papua New Guinea, Vanuatu. Significance was denoted as *** (p<0.001), ** (p<0.01), * (p<0.05).

Table S6. Individual hygiene practices and their association with Health, Hygiene and Safety Development in children by moderators

	Wash hands before a meal			Wash hands after going to the toilet			Tooth brushing		
	Never	Sometimes	Always	Never	Sometimes	Always	Never	Sometimes	Always
	PR (95% CI)	PR (95% CI)	PR (95% CI)	PR (95% CI)	PR (95% CI)	PR (95% CI)	PR (95% CI)	PR (95% CI)	PR (95% CI)
**Age**									
< median	1.22 (1.01; 1.48)*	1.25 (1.06; 1.47)**	1.00 (Ref.)	1.31 (1.09; 1.59)**	1.25 (1.06; 1.47)**	1.00 (Ref.)	1.15 (0.99; 1.35)	0.94 (0.81; 1.10)	1.00 (Ref.)
≥ median	1.12 (0.92; 1.35)	1.20 (1.04; 1.38)*	1.00 (Ref.)	1.33 (1.11; 1.58)**	1.28 (1.11; 1.48)**	1.00 (Ref.)	1.29 (1.11; 1.50)**	1.04 (0.89; 1.20)	1.00 (Ref.)
$P_{interaction}$	0.184	0.605		0.779	0.951		0.032	0.333	
**Sex**									
Female	1.17 (0.96; 1.42)	1.22 (1.04; 1.43)*	1.00 (Ref.)	1.42 (1.17; 1.72)***	1.34 (1.14; 1.58)***	1.00 (Ref.)	1.37 (1.17; 1.60)***	1.02 (0.87; 1.19)	1.00 (Ref.)
Male	1.15 (0.95; 1.38)	1.22 (1.06; 1.42)**	1.00 (Ref.)	1.23 (1.03; 1.46)*	1.20 (1.04; 1.39)*	1.00 (Ref.)	1.10 (0.94; 1.28)	0.97 (0.84; 1.12)	1.00 (Ref.)
$P_{interaction}$	0.627	0.709		0.149	0.171		0.001	0.310	
**Area**									
Rural	1.26 (1.04; 1.54)*	1.38 (1.17; 1.63)***	1.00 (Ref.)	1.63 (1.34; 1.99)***	1.50 (1.26; 1.80)***	1.00 (Ref.)	1.63 (1.34; 1.99)***	1.26 (1.03; 1.55)*	1.00 (Ref.)
Urban	1.10 (0.91; 1.34)	1.06 (0.92; 1.21)	1.00 (Ref.)	1.07 (0.88; 1.30)	1.08 (0.95; 1.24)	1.00 (Ref.)	0.97 (0.84; 1.11)	0.90 (0.79; 1.02)	1.00 (Ref.)
$P_{interaction}$	0.044	0.011		<0.001	0.003		<0.001	0.010	
**SES**									
< median	1.18 (1.01; 1.36)*	1.20 (1.06; 1.35)**	1.00 (Ref.)	1.39 (1.20; 1.61)***	1.29 (1.14; 1.47)***	1.00 (Ref.)	1.36 (1.19; 1.55)***	1.07 (0.94; 1.23)	1.00 (Ref.)
≥ median	1.15 (0.84; 1.58)	1.32 (1.05; 1.65)*	1.00 (Ref.)	1.21 (0.89; 1.63)	1.23 (1.01; 1.52)*	1.00 (Ref.)	0.99 (0.78; 1.25)	0.91 (0.76; 1.09)	1.00 (Ref.)
$P_{interaction}$	0.724	0.633		0.177	0.526		0.009	0.255	
**Country of origin**									
East Asia	3.74 (1.80; 7.73)***	1.65 (0.87; 3.12)	1.00 (Ref.)	4.19 (2.06; 8.50)***	1.74 (0.92; 3.28)	1.00 (Ref.)	1.18 (0.62; 2.26)	0.90 (0.57; 1.40)	1.00 (Ref.)
Southeast Asia and Pacific Region	1.17 (1.03; 1.34)*	1.20 (1.08; 1.33)**	1.00 (Ref.)	1.28 (1.12; 1.46)***	1.25 (1.13; 1.40)***	1.00 (Ref.)	1.09 (0.98; 1.21)	1.01 (0.91; 1.13)	1.00 (Ref.)
$P_{interaction}$	0.001	0.313		<0.001	0.309		0.311	0.898	

Data presented as prevalence ratio (PR) and 95% CIs for poor development (score <25th centile-age). Children who always washed their hands before meals, or who always washed their hands after going to the bathroom, or those who always brushing their teeth were used as the reference group per each case. For tooth brushing, sometimes means once a day. Analyses were adjusted by age, sex, SES, area, and country of origin when these were not the moderators.

East Asia: China and Mongolia, Southeast Asia and Pacific region: Cambodia, Timor-Leste, Papua New Guinea, Vanuatu. Significance was denoted as *** (p<0.001), ** (p<0.01), * (p<0.05).

Table S7. Combinations of hygiene practices and their association with Cognitive Development in children by moderators

	Never	Rarely	Sometimes	Always
	PR (95% CI)	PR (95% CI)	PR (95% CI)	PR (95% CI)
**Age**				
< median	1.83 (1.34; 2.49)***	1.71 (1.33; 2.20)***	1.69 (1.32; 2.16)***	1.00 (Ref.)
≥ median	1.47 (1.11; 1.95)**	1.56 (1.31; 1.87)***	1.38 (1.15; 1.65)**	1.00 (Ref.)
$P_{interaction}$	0.366	0.770	0.065	
**Sex**				
Female	1.62 (1.22; 2.14)**	1.61 (1.32; 1.97)***	1.48 (1.22; 1.81)***	1.00 (Ref.)
Male	1.57 (1.17; 2.12)**	1.62 (1.30; 2.02)***	1.54 (1.24; 1.92)***	1.00 (Ref.)
$P_{interaction}$	0.587	0.677	0.736	
**Area**				
Rural	2.10 (1.58; 2.80)***	2.19 (1.72; 2.80)***	1.89 (1.48; 2.42)***	1.00 (Ref.)
Urban	1.30 (0.91; 1.88)	1.17 (0.97; 1.42)	1.24 (1.04; 1.48)*	1.00 (Ref.)
$P_{interaction}$	0.037	<0.001	0.004	
**SES**				
< median	1.72 (1.37; 2.16)***	1.74 (1.46; 2.08)***	1.50 (1.26; 1.80)***	1.00 (Ref.)
≥ median	1.47 (0.89; 2.43)	1.30 (0.98; 1.73)	1.48 (1.15; 1.91)**	1.00 (Ref.)
$P_{interaction}$	0.994	0.301	0.985	
**Country of origin**				
East Asia	5.61 (2.73; 11.5)***	3.50 (2.14; 5.72)***	2.57 (1.64; 4.04)***	1.00 (Ref.)
Southeast Asia and Pacific Region	1.43 (1.17; 1.76)**	1.51 (1.30; 1.76)***	1.47 (1.26; 1.71)***	1.00 (Ref.)
$P_{interaction}$	<0.001	<0.001	0.007	

Data presented as prevalence ratio (PR) and 95% CIs for poor development (score <25th centile-age). Children who always washed their hands before meals, after going to the bathroom and brushing their teeth, were used as the reference group. Analyses were adjusted by age, sex, SES, area, and country of origin when these were not the moderators.

East Asia: China and Mongolia, Southeast Asia and Pacific region: Cambodia, Timor-Leste, Papua New Guinea, Vanuatu. Significance was denoted as *** (p<0.001), ** (p<0.01), * (p<0.05).

Table S8. Combinations of hygiene practices and their association with Language Development in children by moderators

	Never	Rarely	Sometimes	Always
	PR (95% CI)	PR (95% CI)	PR (95% CI)	PR (95% CI)
**Age**				
< median	1.58 (1.22; 2.05)**	1.26 (1.01; 1.58)*	1.10 (0.88; 1.38)	1.00 (Ref.)
≥ median	1.61 (1.30; 2.00)***	1.40 (1.18; 1.66)***	1.21 (1.02; 1.44)*	1.00 (Ref.)
$P_{interaction}$	0.600	0.836	0.669	
**Sex**				
Female	1.57 (1.24; 1.98)***	1.35 (1.12; 1.64)**	1.10 (0.91; 1.34)	1.00 (Ref.)
Male	1.57 (1.23; 1.99)***	1.29 (1.06; 1.56)*	1.21 (0.99; 1.47)	1.00 (Ref.)
$P_{interaction}$	0.494	0.376	0.568	
**Area**				
Rural	1.80 (1.44; 2.25)***	1.50 (1.22; 1.83)***	1.25 (1.02; 1.54)*	1.00 (Ref.)
Urban	1.19 (0.83; 1.71)	1.18 (0.97; 1.42)	1.08 (0.90; 1.29)	1.00 (Ref.)
$P_{interaction}$	0.009	0.014	0.209	
**SES**				
< median	1.67 (1.40; 2.00)***	1.38 (1.18; 1.60)***	1.15 (0.98; 1.34)	1.00 (Ref.)
≥ median	1.28 (0.71; 2.29)	1.18 (0.87; 1.61)	1.18 (0.90; 1.54)	1.00 (Ref.)
$P_{interaction}$	0.235	0.133	0.975	
**Country of origin**				
East Asia	23.2 (5.50; 98.0)***	5.61 (1.59; 19.8)**	3.62 (1.10; 12.0)*	1.00 (Ref.)
Southeast Asia and Pacific Region	1.54 (1.31; 1.81)***	1.30 (1.14; 1.48)***	1.15 (1.00; 1.32)	1.00 (Ref.)
$P_{interaction}$	<0.001	0.009	0.047	

Data presented as prevalence ratio (PR) and 95% CIs for poor development (score <25th centile-age). Children who always washed their hands before meals, after going to the bathroom and brushing their teeth, were used as the reference group. Analyses were adjusted by age, sex, SES, area, and country of origin when these were not the moderators.

East Asia: China and Mongolia, Southeast Asia and Pacific region: Cambodia, Timor-Leste, Papua New Guinea, Vanuatu. Significance was denoted as *** (p<0.001), ** (p<0.01), * (p<0.05).

Table S9. Combinations of hygiene practices and their association with Socio-emotional Development in children by moderators

	Never	Rarely	Sometimes	Always
	**PR (95% CI)**	**PR (95% CI)**	**PR (95% CI)**	**PR (95% CI)**
**Age**				
< median	1.52 (1.18; 1.96)**	1.30 (1.06; 1.59)*	1.16 (0.94; 1.42)	1.00 (Ref.)
≥ median	1.67 (1.32; 2.10)***	1.41 (1.18; 1.68)***	1.28 (1.07; 1.53)**	1.00 (Ref.)
$P_{interaction}$	0.353	0.276	0.680	
**Sex**				
Female	1.68 (1.31; 2.16)***	1.52 (1.24; 1.85)***	1.34 (1.10; 1.63)**	1.00 (Ref.)
Male	1.48 (1.17; 1.88)**	1.23 (1.02; 1.48)*	1.13 (0.95; 1.36)	1.00 (Ref.)
$P_{interaction}$	0.211	0.036	0.277	
**Area**				
Rural	1.96 (1.53; 2.50)***	1.67 (1.34; 2.07)***	1.47 (1.18; 1.83)**	1.00 (Ref.)
Urban	1.20 (0.87; 1.67)	1.16 (0.97; 1.39)	1.09 (0.92; 1.28)	1.00 (Ref.)
$P_{interaction}$	0.007	0.002	0.015	
**SES**				
< median	1.72 (1.42; 2.10)***	1.42 (1.21; 1.67)***	1.29 (1.10; 1.52)**	1.00 (Ref.)
≥ median	1.27 (0.79; 2.02)	1.31 (1.01; 1.69)*	1.09 (0.86; 1.38)	1.00 (Ref.)
$P_{interaction}$	0.313	0.572	0.261	
**Country of origin**				
East Asia	5.24 (2.34; 11.7)***	3.56 (2.13; 5.95)***	1.93 (1.18; 3.16)**	1.00 (Ref.)
Southeast Asia and Pacific Region	1.54 (1.30; 1.83)***	1.27 (1.11; 1.46)**	1.26 (1.10; 1.45)**	1.00 (Ref.)
$P_{interaction}$	0.001	<0.001	0.055	

Data presented as prevalence ratio (PR) and 95% CIs for poor development (score <25th centile-age). Children who always washed their hands before meals, after going to the bathroom and brushing their teeth, were used as the reference group. Analyses were adjusted by age, sex, SES, area, and country of origin when these were not the moderators.

East Asia: China and Mongolia, Southeast Asia and Pacific region: Cambodia, Timor-Leste, Papua New Guinea, Vanuatu. Significance was denoted as *** (p<0.001), ** (p<0.01), * (p<0.05).

Table S10. Combinations of hygiene practices and their association with Health, Hygiene and Safety Development in children by moderators

	Never	Rarely	Sometimes	Always
	**PR (95% CI)**	**PR (95% CI)**	**PR (95% CI)**	**PR (95% CI)**
**Age**				
< median	1.57 (1.22; 2.03)**	1.38 (1.12; 1.70)**	1.36 (1.11; 1.67)**	1.00 (Ref.)
≥ median	1.44 (1.12; 1.85)**	1.38 (1.16; 1.64)***	1.22 (1.02; 1.44)*	1.00 (Ref.)
$P_{interaction}$	0.558	0.886	0.244	
**Sex**				
Female	1.62 (1.26; 2.10)***	1.51 (1.24; 1.84)***	1.32 (1.08; 1.60)**	1.00 (Ref.)
Male	1.37 (1.07; 1.75)*	1.26 (1.05; 1.51)*	1.26 (1.06; 1.50)*	1.00 (Ref.)
$P_{interaction}$	0.137	0.043	0.770	
**Area**				
Rural	1.92 (1.49; 2.49)***	1.79 (1.43; 2.24)***	1.54 (1.22; 1.94)***	1.00 (Ref.)
Urban	1.06 (0.76; 1.47)	1.09 (0.92; 1.28)	1.10 (0.95; 1.29)	1.00 (Ref.)
$P_{interaction}$	0.001	<0.001	0.016	
**SES**				
< median	1.61 (1.32; 1.95)***	1.45 (1.25; 1.70)***	1.31 (1.12; 1.53)**	1.00 (Ref.)
≥ median	1.15 (0.67; 1.98)	1.26 (0.96; 1.65)	1.25 (0.98; 1.58)	1.00 (Ref.)
$P_{interaction}$	0.202	0.147	0.607	
**Country of origin**				
East Asia	6.19 (2.34; 16.4)***	2.15 (1.09; 4.22)*	1.26 (0.69; 2.30)	1.00 (Ref.)
Southeast Asia and Pacific Region	1.40 (1.17; 1.67)***	1.30 (1.14; 1.48)***	1.33 (1.16; 1.51)***	1.00 (Ref.)
$P_{interaction}$	0.002	0.051	0.918	

Data presented as prevalence ratio (PR) and 95% CIs for poor development (score <25th centile-age). Children who always washed their hands before meals, after going to the bathroom and brushing their teeth, were used as the reference group. Analyses were adjusted by age, sex, SES, area, and country of origin when these were not the moderators.

East Asia: China and Mongolia, Southeast Asia and Pacific region: Cambodia, Timor-Leste, Papua New Guinea, Vanuatu. Significance was denoted as *** (p<0.001), ** (p<0.01), * (p<0.05).

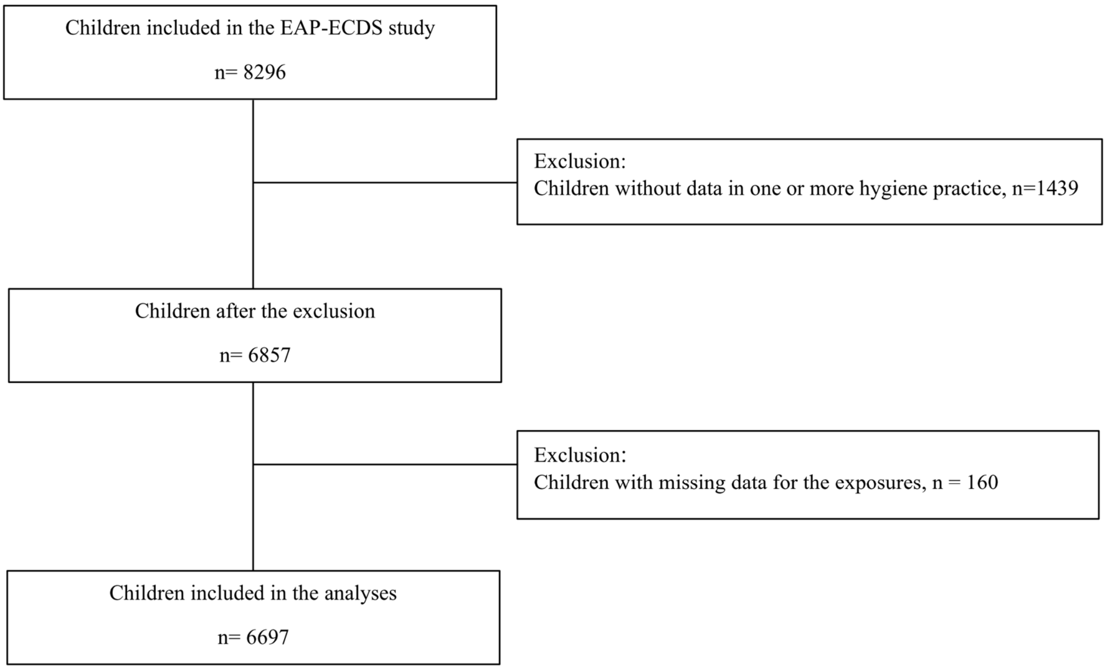

Figure S1. Diagram children included in the study.

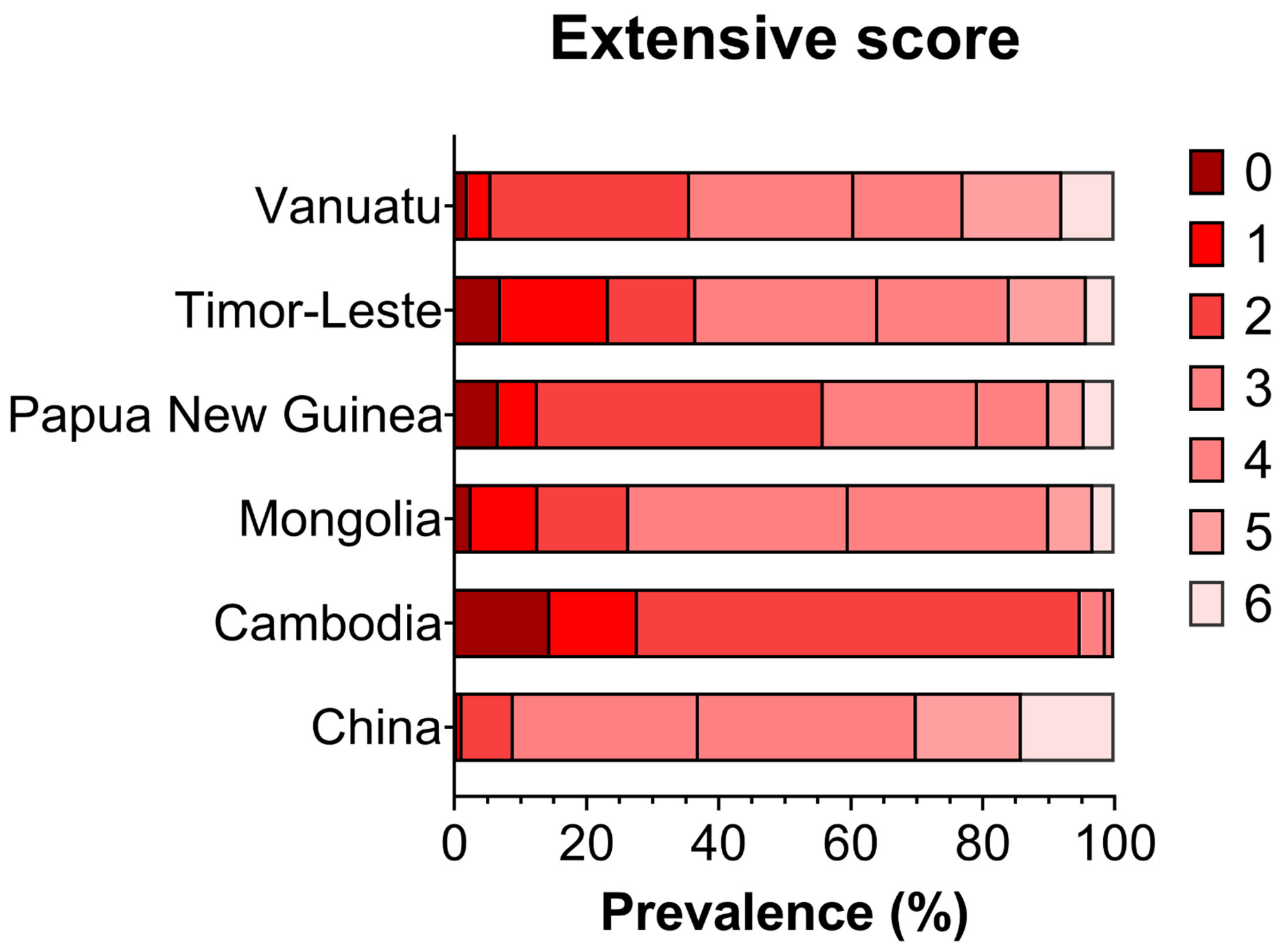

Figure S2. Prevalence of combined hygiene practice by country of origin.
